# Implementing a Statewide Prehospital Sepsis Protocol: Perspectives of Emergency Medical Services Medical Directors

**DOI:** 10.7759/cureus.10781

**Published:** 2020-10-03

**Authors:** Eric Garfinkel, Makoto Tanigawa, Asa Margolis, Matthew J Levy

**Affiliations:** 1 Department of Emergency Medicine, Johns Hopkins University School of Medicine, Baltimore, USA; 2 Department of Emergency Medicine, Thomas Jefferson University, Philadelphia, USA; 3 Office of the Medical Director, Howard County Department of Fire and Rescue Services, Mariottsville, USA

**Keywords:** prehospital, ems, sepsis, medical directors, protocol

## Abstract

Background

Sepsis is a medical emergency that requires prompt recognition and treatment. Multiple Emergency Medical Services (EMS) agencies across the United States have implemented sepsis protocols. In 2016, Maryland instituted its own state-wide EMS sepsis protocol which includes fluid resuscitation, vasopressor administration, and requires alerting the hospital of an incoming sepsis patient.

Objective

The purpose of this study was to quantify the perspectives of EMS medical directors in Maryland regarding prehospital sepsis care and to identify challenges encountered during the implementation of the statewide sepsis protocol.

Methods

A 17-question survey was sent to all 24 jurisdictional medical directors in the state of Maryland.

Results

A total of 14 (58%) medical directors responded, representing four of the five EMS regions in the state. More than half (57%) stated sepsis alerting was a priority in their jurisdiction; however, in a listing of eight initiatives, sepsis was the least prioritized. Most (64%) respondents believed their clinicians had adequate training on sepsis. A majority (79%) of medical directors surveyed felt that core measures of sepsis management would be beneficial. The potentially most helpful core measures were the volume of IV fluid administration (92%), true positive sepsis alerts (83%), and cases of failure to activate a sepsis alert (75%). Engagement of field personnel was rated as the biggest challenge for the implementation of a sepsis protocol in general, and lack of a thermometer on EMS units (50%) was the largest hurdle specifically in the 2016 statewide sepsis protocol. Surveyed medical directors (86%) believe the most difficult obstacle to overcome for EMS clinicians in the treatment of sepsis are nonspecific signs and symptoms.

Conclusions

Prehospital sepsis care was viewed to be important amongst the medical directors surveyed. However, significant challenges to implementation of a sepsis protocol and delivery of prehospital sepsis care are perceived by jurisdictional medical directors. Additional investment and dedication to sepsis care will advance prehospital sepsis treatment in Maryland.

## Introduction

The rapid identification and treatment of sepsis is critical to reducing mortality [1-2]. Nearly 40% of septic patients present to the emergency department via Emergency Medical Services (EMS) [3], which places EMS in an opportune position to positively affect patient outcomes. There is increasing recognition of the potential impact that EMS has on early identification of septic patients [4]. This is similar to other time-sensitive diseases such as stroke or myocardial infarction [5-6].

Prehospital sepsis protocols have been implemented by EMS systems across the country to initiate treatment in the field and expedite physician evaluation upon arrival to the emergency department [7-9]. Recognizing the success of these interventions, Maryland’s State EMS agency, Maryland Institute of Emergency Medical Services (MIEMSS), approved a statewide sepsis protocol in 2016.

Although the protocols are statewide, oversight and implementation occur at the county level. Maryland comprises 24 counties, each with its own medical director. This study aimed to identify medical directors’ perspectives on prehospital sepsis care, including the sepsis protocol. These results will allow for the improvement of sepsis care in Maryland and other EMS systems. 

## Materials and methods

A 17-question survey pertaining to EMS sepsis care was emailed to medical directors of all 24 EMS jurisdictions in Maryland (Appendix 1). The survey was sent by the Office of the State Medical Director of MIEMSS on January 7th, 2020. A reminder email was sent to all jurisdictions on February 19th, 2020. The survey was closed and the results were downloaded on March 4th, 2020.

The questionnaire was created using Qualtrics XM (Qualtrics; Provo, Utah, USA). Participants were anonymous and data was de-identified. Data was only accessible to study investigators and was stored on a secure server. Statistical analysis was performed using Microsoft Excel (Microsoft Corporation; Redmond, Washington, USA).

An institutional review board (IRB) exemption was obtained for this study through the Johns Hopkins University School of Medicine (Baltimore, Maryland, USA).

## Results

Participants

Responses were received from 14 of 24 jurisdictions (58%). Of the 14 responses, 11 responses (78%) were from a medical director and one response each from a deputy medical director, associate medical director, and assistant medical director. Region I (Allegany and Garret counties) had a 0% response rate, Region II (Frederick and Washington counties) had a 50% response rate, Region III (Anne Arundel, Baltimore City, Baltimore County, Carroll, Harford, and Howard counties) had a 100% response rate, Region IV (Caroline, Cecil, Dorchester, Kent, Queen Anne’s, Somerset, Talbot, Wicomico, and Worcester counties) had a 44% response rate, and Region V (Calvert, Charles, Montgomery, Prince George’s, and St. Mary’s counties) had a 60% response rate. The majority (57%) of surveyed medical directors led agencies with a high population (>500,000 people).

Priority of initiatives

Participants were asked to assign seven initiatives a number from 0 to 100 with 100 being the most important initiative in their jurisdiction. If an initiative was not being actively pursued, the participant was instructed to select N/A. Cardiac arrest had the highest mean score of 90, followed closely by ST-elevation myocardial infarction (STEMI) and stroke with mean scores of 87 and 86, respectively. Sepsis had the lowest mean score of the seven initiatives with a score of 54. The “other” option was selected twice. One free response was related to trauma and airway management, and the other to the correct level of triage.

All respondents selected stroke and STEMI as active initiatives. Only one jurisdiction did not have an active initiative for sepsis. The majority (86%) of jurisdictions had ongoing initiatives related to cardiac arrest, the opioid epidemic, and active shooter preparedness.

Challenges to implementation of the EMS sepsis protocol

Participants were asked to rate five perceived general challenges in implementing the sepsis protocol. The answer options were from 0 to 100 with 100 being the greatest challenge. The challenge that received the highest average rating was “engagement of field personnel” with a rating of 64. Other challenges were “inadequate time allocation to properly train personnel” (60), “training opportunities/resources for volunteer personnel” (57), and “inability to assess EMS clinician performance” (53). The least challenging, with an average rating of 31, was “leadership buy-in”.

A thermometer was not always present on EMS units in seven (50%) of jurisdictions, which was the most frequently cited challenge specifically within the Maryland sepsis protocol. Next, 36% of respondents indicated that “suspected source of infection” is not defined well enough in the protocols. Other challenges that participants reported were initiating appropriate treatment when sepsis is suspected (29%) and notifying the receiving hospital that sepsis is suspected (21%). The “other” option was selected twice. Free text answers were regarding the changing definitions of diagnosis, lack of lactate measurement, and no provisions for prehospital antibiotic administration.

Each respondent was asked their opinion of the challenges facing EMS providers regarding prehospital sepsis care. Nearly all (86%) believed that non-specific signs and symptoms were the greatest barrier. Short transport times (57%), general lack of sepsis knowledge (36%), and determining a source of infection (36%) also presented difficulties.

Importance of prehospital sepsis alerts

Participants were asked how important prehospital activation of sepsis alerts are for patient care, and 29% described it as important, 43% neutral, and 29% felt that it was not important.

Next, they were asked how strongly they agreed with the statement “sepsis alerting is a priority initiative in my jurisdiction”. Eight of fourteen jurisdictions (57%) somewhat agreed, agreed, or strongly agreed. Four of fourteen jurisdictions (29%) neither agreed nor disagreed. Two of fourteen jurisdictions (14%) somewhat disagreed with the statement. No jurisdictions disagreed or strongly disagreed.

Sepsis core measures

Receiving data regarding core measures would be beneficial in assessing EMS clinician performance according to 11 of 14 respondents (79%). The remaining three respondents (21%) neither agreed nor disagreed with the statement. No jurisdictions disagreed with the statement.

Participants were asked which core measures they would find useful in assessing EMS clinician performance of prehospital sepsis care. Most jurisdictions reported IV fluid volume administration (92%), true positive sepsis alerts (83%), cases of failure to activate sepsis alerts (75%), number of prehospital sepsis alerts (58%), and false positives (58%) would be useful. Less than half of jurisdictions reported that IV access initiation (42%) or vasopressor administration (25%) would be useful. The “other” option was selected once (8%) and the free-text response was regarding field lactate. Two participants did not answer. 

Follow up from hospitals

Medical directors from six of fourteen jurisdictions (43%) reported that they do not receive any follow-up information from hospitals regarding patients activated as a sepsis alert. Of those that do receive information, five jurisdictions (38%) receive information from some hospitals, and two (14%) receive information from most hospitals.

For jurisdictions that receive follow-up information, four jurisdictions (44%) obtain data via accessing Chesapeake Regional Information System for our Patients (CRISP), the health information exchange for hospitals in Maryland and the District of Columbia. Four (44%) jurisdictions obtain data from hospital liaisons and one jurisdiction (11%) receives information via email request.

EMS providers training on sepsis

Regarding training on sepsis, nine of fourteen jurisdictions (64%) agreed that EMS providers have had adequate training, two out of the fourteen jurisdictions (14%) neither agreed nor disagreed, and three jurisdictions (21%) indicated that EMS providers were inadequately trained. EMS clinicians received sepsis education via in-service training (57%), case reviews (57%), lectures (50%), and simulation (29%).

Receptiveness of EMS clinicians and leadership

EMS clinicians were viewed as moderately, very, or extremely receptive to the implementation of the sepsis protocol by 71% of medical directors. The remaining 29% of medical directors believed their jurisdiction’s EMS clinicians were only slightly or not at all receptive to the sepsis protocol. 

Only 21% of medical directors considered their organizational leadership to be slightly receptive to the implementation of the sepsis protocol. Zero jurisdictions were not at all receptive. Moderately receptive or higher was selected by 79% of medical directors, with 57% answering very or extremely receptive. 

## Discussion

As prehospital sepsis care advances throughout the country, there is a need to understand the viewpoint of EMS medical directors and identify potential barriers to care. On review of current literature, no studies assess these perspectives or challenges. The results of this study are novel in highlighting the complex situations facing Maryland medical directors.

Sepsis was the least prioritized of eight initiatives, falling far behind stroke, STEMI, and cardiac arrest. These diseases have been at the forefront of EMS for the past few decades, with well-established and closely tracked metrics. The Joint Commission and Centers for Medicare & Medicaid Services began collecting measures on acute myocardial infarction from hospitals in 2002 [10], and EMS plays an integral role in shortening door to balloon time. Sepsis core measures are new, established in 2015 [11], and are not as widely reported. However, despite not being a top priority, nearly every (93%) jurisdiction had some form of sepsis initiative in place. This is encouraging and likely reflects the emerging awareness of the importance of sepsis.

Implementation of the 2016 sepsis protocol (Table 1) has produced many challenges for medical directors across Maryland. The majority (79%) of medical directors agree that access to core measures, such as the number of true or false positive sepsis alerts, is important to assess EMS clinician performance. Yet almost half (43%) reported receiving no follow up patient data from local hospitals, and only 14% have the ability to follow up on all sepsis alert patients. Difficulties in assessing the performance of EMS clinicians was noted to be a challenge to implementing the protocol for 71% of respondents. Fortunately, access to Maryland’s health information exchange is now available to all Maryland EMS medical directors.

**Table 1:** Maryland State Sepsis Protocol

Maryland State Sepsis Protocol
Any patient with a suspected source of infection PLUS at least two of the following meets sepsis criteria: 1) Temperature greater than 100.4^○ ^Fahrenheit or less than 95.9^○ ^Fahrenheit 2) Heart rate greater than 100 beats per minute 3) Respiratory rate greater than 25 (or End Tidal CO_2_ less than or equal to 32mmHg) or 4) Hypotension (systolic blood pressure less than 90mmHg)
Hypotensive patients should receive fluid resuscitation, up to 30cc/kg. If refractory to fluids, an epinephrine drip is initiated.
The EMS clinician is required to notify the receiving hospital of a SEPSIS ALERT if the patient meets sepsis criteria

The sepsis alert portion of the protocol did not have significant support. Only slightly more than a quarter (29%) of medical directors responded that they are important for patient care. This finding is consistent with emergency medicine literature that reports 76% of emergency department providers do not find an early warning system improves patient care, despite a change in patient management occurring 44% of the time such as closer monitoring or additional interventions [12]. The lukewarm support for sepsis alerts appears to extend to sepsis care in general as evidenced by 50% of EMS units lacking a thermometer, which is a necessity to evaluate for infection. Of note, it would be curious to evaluate how, if at all, this accessibility of thermometers might have changed in the setting of the coronavirus disease (COVID-19) pandemic. 

Although every jurisdiction reported that there is some form of additional training or education regarding prehospital sepsis care, only 64% believed that EMS clinicians have received adequate training. Identification of sepsis by prehospital clinicians was seen as a challenge by 86% of medical directors due to non-specific signs and symptoms. This may be addressed with targeted training towards sepsis. Additional sepsis education may also improve EMS clinician engagement in using the sepsis protocol, which was ranked as the top issue for protocol implementation.

The solution to these challenges lies in viewing sepsis as equal to other time-sensitive, high-consequence conditions. Prioritization of sepsis on the same level as STEMI and stroke would result in enhancing continuity of care between hospitals and EMS systems, as well as increasing time for sepsis education and additional investment in equipment. Front line EMS clinicians (93%) and organizational leadership (100%) are receptive towards a sepsis protocol, which bodes well for future sepsis initiatives.

Limitations

This study has several limitations. Jurisdictional medical directors are a critical component of the protocol creation process, and it is very likely that many of the surveyed medical directors contributed to the creation of the sepsis protocol. This may have introduced bias and affected their answers to the questions. Additionally, the low response rate and the disproportionate amount of representation from urban jurisdictions may limit the ability of these results to be applied to rural EMS systems. The data was limited to sepsis care within Maryland. Finally, given the format, this study suffers from potential selection bias.

## Conclusions

Medical Directors are essential in setting the tone for the clinical priorities of an EMS agency. This analysis revealed that medical directors must balance multiple competing high-priority initiatives as well as several logistical, operational, and human factors. These issues create several barriers with regards to implementing an EMS sepsis program. An ongoing and increased commitment to sepsis care by EMS jurisdictions is required to continue to advance prehospital sepsis management.

## Appendices

Appendix 1: Survey questions

1. Please indicate which best describes your role within your EMS jurisdiction. If you are part of multiple EMS agencies, please indicate your highest role. 1) Medical director 2) Deputy medical director 3) Associate medical director 4) Assistant medical director

2. Please indicate the region of your EMS jurisdiction. 1) Region I 2) Region II 3) Region III 4) Region IV 5) Region V

3. Please indicate the size of the population served by your jurisdiction. 1) >500,000 people 2) 250,000 to <500,000 people 3) 125,000 to <250,000 people 4) 75,000 to <125,000 people 5) <75,000 people

4. Please rate how important you believe prehospital activation of sepsis alerts is for patient care from most important (100) to least important (-100).

5. Please indicate how strongly you agree with the following statement: Sepsis alerting is a priority initiative in my jurisdiction. 1) Strongly disagree 2) Disagree 3) Somewhat disagree 4) Neither agree nor disagree 5) Somewhat agree 6) Agree 7) Strongly agree

6. Please rate, by priority, the following initiatives that your jurisdiction may be currently focusing on, with 100 being of the most importance. If your jurisdiction is not working on a given initiative, please indicate N/A. 1) STEMI 2) Stroke 3) Cardiac Arrest 4) Sepsis 5) Opioid Epidemic/Naloxone 6) Mobile Integrated Community Health 7) Active Shooter Preparedness 8) Other

7. Please indicate how strongly you agree with the following statement:  EMS clinicians in my jurisdiction have had adequate training on the prehospital emergency care for sepsis. 1) Strongly disagree 2) Disagree 3) Somewhat disagree 4) Neither agree nor disagree 5) Somewhat agree 6) Agree 7) Strongly agree

8. Please indicate any additional training/education that has been provided to EMS clinicians in your jurisdiction, beyond the MIEMSS protocol update, regarding prehospital emergency care for sepsis. 1) Lecture 2) In-service training 3) Simulation training 4) Case reviews 5) Other

9. Please indicate how strongly you agree with the following statement: Having data of core measures regarding sepsis management would help better assess EMS clinician performance with prehospital emergency care for sepsis. 1) Strongly disagree 2) Disagree 3) Somewhat disagree 4) Neither agree nor disagree 5) Somewhat agree 6) Agree 7) Strongly agree

10. Please indicate which core measures you think would be most useful in assessing EMS clinician performance related to prehospital sepsis care. 1) Number of prehospital sepsis alerts 2) IV access initiation 3) IV fluid volume administered 4) Vasopressor administration 5) True positive (appropriate sepsis alert activation) 6) False positives (false activations) 7) Cases of failure to activate 8) Other

11. Does your jurisdiction receive sepsis follow-up information/outcomes from the hospitals that your EMS clinicians routinely transport to? 1) Yes, most hospitals 2) Yes, some hospitals 3) No

12. If your Answer to the previous question was Yes, please indicate how this information is currently obtained from hospitals. If your answer was no, please select N/A. 1) Access via CRISP 2) Direct liaison from hospital 3) Other

13. Please rate the following challenges you have faced in implementing the Sepsis Protocol in your jurisdiction with 0 being not a challenge, and 100 being the greatest challenge, or N/A if not a challenge. 1) Leadership buy-in 2) Engagement of field personnel 3) Inadequate time allocation to properly train personnel (both career and volunteer) 4) Training opportunities/resources for volunteer personnel specifically 5) Inability to assess EMS clinician performance 6) Other more urgent organizational priorities

14. Please indicate any particular elements of the Sepsis Protocol that have been most challenging to implement. 1) “Suspected source of infection” not defined well enough in protocols 2) Thermometer not always available on EMS units 3) Notification to hospital when sepsis is suspected 4) Initiation of appropriate treatment when sepsis is suspected 5) Other

15. In your opinion, what are the greatest challenges that EMS clinicians have related to prehospital sepsis care. 1) General lack of knowledge of sepsis 2) Non-specific signs and symptoms 3) Short transport times 4) Determining a “suspected source of infection” 5) Other

16. Please rate how receptive your front-line EMS clinicians have been to the implementation of the Sepsis Protocol. 1) Not at all receptive 2) Slightly receptive 3) Moderately receptive 4) Very receptive 5) Extremely receptive

17. Please rate how receptive your organization's leadership have been to the implementation of the Sepsis Protocol. 1) Not at all receptive 2) Slightly receptive 3) Moderately receptive 4) Very receptive 5) Extremely receptive
